# Frequency and Antimicrobial Susceptibility Patterns of Diabetic Foot Infection of Patients from Bandar Abbas District, Southern Iran

**DOI:** 10.1155/2020/1057167

**Published:** 2020-06-09

**Authors:** Arman Ahmadishooli, Parivash Davoodian, Saeed Shoja, Bita Ahmadishooli, Habib Dadvand, Hosein Hamadiyan, Reza Shahriarirad

**Affiliations:** ^1^Infectious and Tropical Diseases Research Center, Hormozgan Health Institute, Hormozgan University of Medical Sciences, Bandar Abbas, Iran; ^2^Student Research Committee, Hormozgan University of Medical Sciences, Bandar Abbas, Iran; ^3^Molecular Medicine Research Center, Hormozgan University of Medical Sciences, Bandar Abbas, Iran; ^4^Thoracic and Vascular Surgery Research Center, Shiraz University of Medical Science, Shiraz, Iran; ^5^Student Research Committee, Shiraz University of Medical Sciences, Shiraz, Iran

## Abstract

Diabetic foot infection is among the most common complications of diabetes mellitus which significantly causes hospitalization and is the most prevalent etiology of nontraumatic amputation worldwide. The current study aimed at assessing the frequency and antimicrobial susceptibility patterns of diabetic foot infection of patients from the Bandar Abbas area, in the south of Iran. In this study, a total of 83 diabetic patients with diabetic infected foot wounds referring to Shahid Mohammadi Hospital, Bandar Abbas, from 2017 to 2018 were assessed. Samples were obtained from wound sites and evaluated by aerobic culture and also an antibiogram test for antibiotic susceptibility. Factors including age, sex, type of diabetes, the medication used for diabetes, previous history of diabetic foot infection, duration of wound incidence, fever, and laboratory indices were recorded for each subject. The most prevalent detected bacteria were *Escherichia coli* (20.5%), *Enterococcus sp.* (16.9%), *Klebsiella sp.* (12%), *Staphylococcus aureus* (8.4%), *Enterobacter sp.* (7.2%), and *Acinetobacter sp.* (6%). The results of antibiogram tests revealed the most and the least antibiotic sensitivity for *E. coli sp.* as meropenem and ciprofloxacin, for *Enterococcus sp.* as gentamicin and ciprofloxacin, for *Klebsiella sp.* as amikacin and cotrimoxazole, and for *Enterobacter sp.* as cotrimoxazole and both amikacin and ciprofloxacin. *Staphylococcus aureus* was sensitive to vancomycin and doxycycline, and *Acinetobacter sp*. was 100% resistant to all antibiotics except amikacin and gentamycin. A significant statistical association was found between the C-reactive protein and the patients' diabetic foot infection organisms (*P*=0.019). Findings of the study revealed *E. coli sp.* as the most common bacteria which are infecting the foot lesions in the studied population. The highest antibiotic susceptibility was seen for vancomycin, linezolid, and carbapenem.

## 1. Introduction

According to 2017 statistics, 425 million people worldwide have diabetes. Compared to 2013 and 1980, in which reported 382 million and 108 million, respectively, it can be seen that this progressive chronic metabolic disease is developing rapidly worldwide [1, 2].

The major complications of this disease are in the form of microvascular and macrovascular complications, in which sedimentation and accumulation of glucose and related metabolites in the vessels chronically decrease blood supply and cause damage to the tissue [3]. Diabetic ulcers develop due to poor blood supply following diabetes. The most common site of diabetic ulcers is the foot. Although other areas of the body are prone to such ulcers, for some reasons including neuropathy as the main etiology, neglecting this part of the body, the shape of the arch and toes, and the colonization of bacteria and fungi between the toes due to the sweating of the foot in the socks are all causes of this complication, mainly in the toes [4–6]. Classical diabetic foot ulcers are mainly in the form of chronic, small, midpunctured wounds and usually on the plantar surface on deformed metatarsals and Charcot's joints [7].

These wounds carry a heavy psychological and financial burden on the patients' family and the community health system. However, maintaining health and attention can easily minimize this effect, and effective preventive measures can remove the cost of the medical and psychological burden from diabetics and the community. The importance of this is better understood when knowing that diabetic foot ulcers will recur in more than 50% of cases over the next 3 years [8, 9]. In 2005 the International Diabetes Federation has attributed their concentration on the global burden of diabetic foot disease. The lifetime risk of a diabetic patient developing a foot ulcer could be as high as 25% [2] and it is estimated that every 30 seconds, a lower limb is lost as a consequence of diabetes worldwide [10]. Over 20% of the cases of hospitalization due to diabetes are due to diabetic foot ulcers. These wounds can lead to organ damage or even deadly and dangerous infections for patients. Therefore, the need for antibiotic treatment to minimize these complications is of great importance [11, 12].

It is not uncommon for a diabetic foot ulcer to be treated incorrectly. This is especially due to the lack of specialized diabetic foot ulcer treatment centers. Mistreatment of diabetic foot ulcers can be caused by factors such as the use of antibiotics without sensitivity in culture or drugs that do not affect the species extracted from the wound site or incorrect duration of treatment [13].

Most acute infections in patients who have not been treated with antibiotics are mostly monobacterial and occur, at least in western countries, predominantly with aerobic Gram-positive cocci (especially *Staphylococcus aureus*). Infections that are chronic or have a previous history of antibiotic treatment are often polymicrobial, generally occurring with Gram-positive aerobic cocci or obligate anaerobe Gram-negative bacilli [14].

Previous studies have shown Gram-positive aerobic coccyx bacteria, mainly *Staphylococcus aureus*, are the most common causes of diabetic foot ulcer infection. In chronic ulcers, especially those that have recently been treated with antibiotics, infections are mainly polymicrobial. The pathogens in these infections take quite different forms as they are often caused by Gram-negative aerobic bacilli and compulsive anaerobic bacteria [15]. The presence of polymicrobial patterns in these wounds results in the interaction of bacterial factors and the production of virulent factors such as hemolysin, proteases, and collagenases. Short-chain fatty acids are also produced. These factors cause inflammation, delayed wound healing, and, ultimately, severe chronic ulcers [16].

In chronic noninfectious wounds, the colonization of some microbes is likely to induce passive resistance. Even studies have shown that chronic noninfectious wounds are the site of colonization of germs that were not even previously found in any study [17]. Also, in some studies, mainly in developing countries, microbes isolated from noninfectious diabetic foot ulcers originate largely from aerobic Gram-negative bacilli, especially *Pseudomonas aeruginosa* [18].

Given that the pattern of bacterial susceptibility to different types of antibiotics varies from region to region, and the necessity of choosing an antibiotic treatment to maximize treatment response and minimize bacterial resistance [19], the present study evaluates the susceptibility pattern of diabetic foot ulcer infection to Shahid Mohammadi Hospital in Bandar Abbas, in the south of Iran.

## 2. Materials and Methods

In this study, after reviewing the database for patients referring to Shahid Mohammadi Hospital, Bandar Abbas, in the south of Iran in 2017–2018 with the impression of diabetic foot ulcer, records of these patients were selected and patients with documented culture results of the pathogen of diabetic foot ulcer were included in this study. A total of 300 diabetic foot wound infection patients were assessed in which 83 cases with foot lesions were enrolled with documented results of the causative pathogen of diabetic foot ulcers. Samples were obtained from wound sites, before starting antibiotic treatment, through biopsy specimens from deep tissues, and, if there was a purulent discharge, specimens were prepared using syringes or swabs. The samples were placed in a sterile container and transferred to the laboratory of Shahid Mohammadi Hospital for aerobic culture and also antibiogram test for antibiotic susceptibility. The bacteria were evaluated for antimicrobial susceptibility tests based on a study by Humphries et al. [20]. For Gram-positive bacteria including *Staphylococcus aureus* and *Streptococcus sp.*, antibiotics including oxacillin, clindamycin, cefalexin, levofloxacin, amoxicillin-clavulanic acid, doxycycline, trimethoprim, sulfamethoxazole, vancomycin, and daptomycin were tested, and for Gram-negative rods, antibiotics including cefoxitin, ceftriaxone, ampicillin, sulbactam, tigecycline, ciprofloxacin, imipenem, and gentamicin, and for *Pseudomonas aeruginosa*, piperacillin-tazobactam, ceftazidime, cefepime, tigecycline, ciprofloxacin, imipenem, and gentamycin were tested. The antibiotic susceptibility of the bacteria was determined by the CLSI guidelines [6].

Moreover, factors including demographic information such as age, sex, type of diabetes and treatment, duration of disease, previous history of diabetic foot infection, duration of wound incidence, fever, erythrocyte sedimentation rate (ESR), C-reactive protein (CRP), fasting blood sugar (FBS), hemoglobin A1C, type of treatment, smoking, leukocytosis, and previous history of amputation were also recorded.

Data were analyzed by SPSS (version 19) software. Descriptive statistics were used as mean and percentage and chi-square test was used for analysis. A *P* value of less than 0.05 was considered significant.

## 3. Results

The mean age of patients was 56.86 (SD = 13.07) years, the minimum age was 26 years, and the highest was 87 years. The highest frequency (34.9%) was in the age group above 60 years. Out of 83 patients, 50 (60.2%) were male and 33 (39.8%) were female. The duration the patient suffered from diabetes ranged from 1 to 30 years (mean = 12.4, SD = 7.96) with the highest frequency in the group under 10 years. Of the 83 patients, 27 (32.9%) used to receive oral medicine and 55 (67.1%) used insulin for the control of diabetes.

The FBS level of the patients ranged from 90 to 430 (mean = 209.66; SD = 72.572) with the highest frequency in the FBS group under 126 with 39 cases (47%).

Of the studied patients, 37 (67.3%) reported having previous diabetic ulcers while 18 (32.7%) had no previous history of diabetic ulcers. Also, 28 (33.7%) had a history of a previous amputation due to a diabetic ulcer.

The ESR levels of the patients ranged from 14 to 148 (mean = 78.58; SD = 31.62). Also, 57 (78.1%) of the patients had positive CRP and 41 (50.6%) of the patients had leukocytosis.

The most isolated bacteria were *E. coli* (20.5%) followed by *Enterococci* (16.9%) and *Klebsiella* (12%).  Table 1 shows the isolated bacteria from the diabetic foot infection of patients.

Also, a number of cases were reported in diabetic foot wounds in where more than one pathogen has been reported, which included *Staphylococcus aureus w*ith *Acinetobacter sp.*, *Enterobacter sp.* with *Acinetobacter sp.*, *Klebsiella sp.* with *enterococci sp.*, *Klebsiella sp.* with *Enterobacter sp.*, *Staphylococcus aureus* with *Streptococcus beta-hemolytic sp.*, *Enterococcus sp.* and *Pseudomonas sp.*, *Proteus vulgaris.* and *Acinetobacter sp.*, *E. coli sp.*, *Klebsiella sp.*, and *Proteus mirabilis*, *E. coli sp.* with *Candida sp.*, *Enterococcus sp.* with *Streptococcus viridans*, *Enterococcus sp.* with *E. coli sp.*, *Enterococcus sp.* with *Klebsiella sp.*, *Staphylococcus aureus* with *E. coli*, and *E. coli* and *Enterobacter sp*. Therefore, based on these results, *E. coli sp* and *Enterococcus sp.* were the most dominant pathogen in polymicrobial infection (5 cases), followed by *Klebsiella sp.* (4 cases) and *Staphylococcus aureus* (3 cases).

There was no significant association between the infecting organism and the patients' age or gender (*P* value = 0.810 and 0.533, respectively). Also there was no association between the patients' FBS, ESR, HbA1c, leukocytosis, and fever with the infecting organism (*P* value = 0.367, 0.729, 0.506, 0.231, and 0.415, respectively). Likewise, there was no correlation between the infecting pathogen in diabetic foot ulcer with the patient's history of smoking, previous history of diabetic ulcer, type of medication for diabetes (oral or insulin), duration of the ulcer, duration of diabetic disease, or history of amputation (*P* value = 0.750, 0.268, 0.355, 0.464, 0.253, and 0.509). However, our results showed a significant association between the patients' CRP and the infecting organism (*P*=0.019).

The results of antibiogram tests revealed the most and the least antibiotic sensitivity for *E. coli sp.* as meropenem and ciprofloxacin, for *Enterococcus sp.* as gentamicin and ciprofloxacin, for *Klebsiella sp.* as amikacin and cotrimoxazole, and for *Enterobacter sp.* as cotrimoxazole and both amikacin and ciprofloxacin. *Staphylococcus aureus* was sensitive to vancomycin and doxycycline, and *Acinetobacter sp*. was 100% resistant to all antibiotics except amikacin and gentamycin. Tables 2 and 3 show the sensitivity pattern of Gram-negative organisms isolated from infected diabetic foot ulcer, and Tables 4 and 5 show the sensitivity pattern of Gram-positive organisms isolated from infected diabetic foot ulcers.

Furthermore, regarding methicillin-resistant *Staphylococcus aureus* (MRSA), our results and based on Table 4, 2 out of 6 (33%) Staphylococcus were resistant to oxacillin and cefoxitin.

## 4. Discussion

Diabetic foot ulcer accounts for 20% of hospitalizations due to diabetes mellitus, with the highest rate of hospitalization due to diabetes. In many cases, diabetic foot ulcers are even up to 50% likely to be infected. This infection can manifest itself as cellulite, osteomyelitis, abscess, tendonitis, septic arthritis, and necrotizing fasciitis [21–23]. As a result, appropriate antibiotic treatment for diabetic wound infections is of particular importance and shows its peak effect in the first 72 hours [24]. The importance of our study becomes clearer when it is aimed at evaluating microbial ulcers and the use of narrow-spectrum antibiotics instead of the broad-spectrum use of antibiotics [25]. Due to regional differences, unnecessary use, and nonregular use in different areas, antibiotic resistance has been developed and makes proper treatment difficult [26].

In this study, 83 diabetic patients referred to Shahid Mohammadi Medical Center for diabetic foot ulcers were evaluated for microorganisms, antibiotic susceptibility, and related factors. The findings of this study showed that the most common microorganisms extracted from wounds were *E. coli sp.*, followed by *Enterococcus sp.*, *Klebsiella sp.*, *Staphylococcus aureus*, and *Enterobacter sp.*, respectively. *Citrobacter sp.*, *Flavobacter sp.*, *Staphylococcus beta-hemolytic sp.*, and *Streptococcus beta-hemolytic sp.* were the least strains extracted from diabetic foot ulcers. A study by Rampal et al. [27] has shown that Gram-negative bacteria was predominant in DFI compared to Gram-positive bacteria (71 versus 29%); however, in their study, the most dominant microorganism was *Pseudomonas aeruginosa*, followed by *Proteus mirabilis* and *Klebsiella sp*. for Gram-negative isolates and Gram-positive isolates consist of *Staphylococcus aureus* followed by *Streptococcus sp*. Our results are also supported by previous studies in Malaysia, India, and Turkey that recorded similar observations where Gram-negative bacteria predominate in diabetic foot infection [28, 29]. In contrast to that, studies from the western countries showed more diabetic foot infection caused by Gram-positive bacteria [30, 31]. A theory proposed by Ramakant et al. for the difference in the nature of microbes infecting the diabetic foot infection has been due to variation in environmental factors such as sanitary habits, e.g., use of water for perianal wash (ablution) after defecation that can often lead to contamination of hands with fecal flora that is rich in Gram-negative bacteria [32].

The overuse and abuse of the antimicrobial drugs can contribute to the wide spreading of multidrug resistant (MDR) microorganisms [33]. The differences of MDR bacteria in diabetic foot infection might be due to various factors, such as the demographic, age, sex, ulcer assessments, diabetic glycemia control, and duration of hospitalization and former use of antibiotics management. Furthermore, the hospitalization might considerably disturb the presence and type of MDR organisms on diabetic foot ulcer, where patients are subjected to cross-infection by the colonization of nosocomial pathogens that resist most prescribed antibiotics and might be skin commensal [34].

In a study by Jneid et al., in 2018, the most extracted organism from diabetic foot ulcer tissue was *Staphylococcus aureus sp.*, followed by *Enterococcus faecalis*, *Enterobacter cloacae*, *Staphylococcus lugdunensis*, *Proteus mirabilis*, *Staphylococcus epidermidis sp.*, and *Finegoldia magna.* They also established that after a 1-month follow-up, the only factor related to wound improvement was the presence of *E. faecalis*, compared to patients without wound improvement [35].

The common colonization with *Staphylococcus aureus* has also been reported in other studies [36, 37]. A study in France also evaluated the bacterial agents of diabetic foot ulcers and infection in which *Staphylococcus aureus* was also the leading cause of diabetic foot infections. The point highlighted in these studies is the virulence over this bacterial agent that has led to significant bacterial resistance [38–40]. In a recent study in 2019, Pitocco et al. reported the microorganism causing diabetic foot ulceration, respectively, *Staphylococcus aureus*, *Enterococcus faecalis*, and *Pseudomonas aeruginosa* [41].

Our study showed 14 types of polymicrobial infection out of total 83 samples collected from infected diabetic foot ulcer cases. Also, the most dominant pathogen in polymicrobial infection was *E. coli sp.* and *Enterococcus sp.* The present findings are supported by previous studies, which found the dominance of monomicrobial infections [42] Hassan et al. reported a predominance of monomicrobial infections (77.3%), while polymicrobial infections were found in 22.7% [34]. However, Saseedharan et al. reported [43] higher frequencies of polymicrobial than monomicrobial infections which can be clarified by the circumstance that most studies rely on exploration of microbiologist through isolation of the normal microbial flora and the pathogenic isolates deprived of concern of patient's history, particularly the prior antibiotics scenario.

Our study showed no significant correlation between the causing organism of the diabetic foot ulcer and the patient's type of diabetes, duration of diabetes, duration of the wound, history of amputation, and smoking. A study by Peters et al. on factors contributing to ulcer complications in 2005 reported that a history of previous ulcers, duration of more than 30 days, trauma as a cause of ulcers, and peripheral vascular disease are associated with poor response to antibiotic therapy [44]. Also, in our study, history of previous diabetic foot ulcer was another factor that did not show any significant relation with pathogen type. However, other studies have shown that previous ulcers associated with unusual Gram-negative and anaerobic pathogen infections are causes of poorer prognosis in infected diabetic foot ulcers [45, 46].

There was also no significant correlation between pathogen of infected diabetic foot ulcer and the patients' ESR, FBS, HbA1c, fever, and leukocytosis. However, there was a significant correlation between the patients' positive CRP level and the causing organism (*P*=0.019). Many studies have focused on the role of inflammatory markers to predict the onset of inflammation and infection, especially in bacterial involvement of diabetic wounds [47, 48]. It has also been shown that in patients with high ESR levels, CRP aids to distinguish osteomyelitis from soft tissue infection [49].

Our study showed no significant correlation between the patients' age group and the infecting organism. Previous studies regarding the patients' age showed worse prognosis in younger ages [50]. Lavery et al. in their study noted that younger people were at higher risk of developing osteomyelitis as one of the most serious complications of diabetic foot ulcers [51].

A study by Jia et al. regarding the patients' sex and prognosis of diabetic foot ulcer infection reported female sex as a worsening prognosis factor [52]. Other studies have also reported a higher risk of infection of diabetic foot ulcers in females [45]. However, our study showed no significant correlation between the two genders.

Our study also evaluated the sensitivity of different pathogens to antibiotics. The most common antibiotics tested were vancomycin and gentamicin, which showed more than 80% sensitivity to enterococci. Linezolid was also 75% susceptible to enterococcal pathogens.

In a study by Demetriou et al., they examined the extent of bacterial resistance to antibiotics. In their study, the lowest drug resistance was observed in piperacillin-tazobactam and they also reported a good therapeutic response to all anti-*Staphylococcus aureus* drugs. The highest resistance in their study was towards clindamycin, erythromycin, and amoxicillin-clavulanate [53].

Al Benwan et al. reported the highest Gram-negative responses to piperacillin-tazobactam, amikacin, and imipenem [54]. A study by Sekhar et al. reported the highest therapeutic response of Gram-negative strains was observed towards amikacin, cefoperazone-sulbactam, and meropenem. They also reported a 100% response to *Staphylococcus aureus* to cotrimoxazole and 100% resistance to ciprofloxacin [55]. However, other studies have shown to some extent the sensitivity of this common strain to ciprofloxacin [17]. In the studies of Bansal et al. and Gadepalli et al. in *methicillin-resistant Staphylococcus aureus* (MRSA) cases, antibiotic susceptibility to cotrimoxazole, linezolid, and doxycycline was found. Noteworthy in these studies was the remarkable resistance to vancomycin as the main antistaphylococcal drug [17, 28].

In the study of Perim et al., the highest sensitivity to both Gram-negative and Gram-positive strains was found in meropenem. MRSA bacteria, of course, responded well to vancomycin, although some resistance was also found. Also, gentamicin was one of the drugs with an appropriate therapeutic response to a Gram-negative diabetic foot ulcer. However, anaerobic bacteria were not included in their study [56].

In the study of Rastogi et al., an appropriate therapeutic response to quinolones, third-generation cephalosporins, and carbapenems was found in *Pseudomonas aeruginosa* strains. This study reported 100% sensitivity to vancomycin in the evaluation of Gram-positive, including *Enterococci sp.* as well as *Staphylococcus aureus* [26].

Limitations include the inability to generalize the study to whole Iran as it was mainly concentrated on the study location. Also, due to the retrospective study design, evaluating the clinical presentation of the patients was not possible along with the lack of documented information in hospital records. Also, indication of the used classes of the empirical antibiotics over the antibiotic susceptibility test, similar to the one described by Hassan et al. [34], could be beneficial in designing empirical treatment protocols in diabetic foot infection patients. Further prospective studies are required to evaluate the clinical features along with the response to antibiotic treatment in diabetic foot infection patients.

## 5. Conclusion

The most common bacteria isolated from the foot ulcers were *E. coli* for Gram-negative and *Enterococci sp.* for Gram-positive. Also, the highest antibiotic susceptibility to vancomycin, linezolid, and carbapenem was observed. This study provided valuable information concerning DFIs in Bandar Abbas, Southern Iran, which might help to prevent further severe complications particularly the amputation of the extremity limbs. Further studies on the organism isolated from infected diabetic foot ulcers in other areas of Iran are justified.

## Figures and Tables

**Table 1:** Isolated bacteria from a diabetic foot infection.

Pathogen	Frequency	Positive (%)
*E. coli*	17	20.5
*Enterococci*	14	16.9
*Klebsiella*	10	12
*Staphylococcus aureus*	7	8.4
*Enterobacter*	6	7.2
*Acinetobacter*	5	6
*Staphylococcus epidermidis*	4	4.8
*Proteus mirabilis*	3	3.6
*Proteus vulgaris*	3	3.6
*Coagulase-negative Staphylococcus aureus*	2	2.4
*Streptococcus viridans*	2	2.4
*Candida*	2	2.4
*Pseudomonas*	2	2.4
*Flavobacterium*	1	1.2
*Streptococcus beta-hemolytic*	1	1.2
*Citrobacter*	1	1.2
No organism	2	2.4

**Table 2:** Sensitivity pattern of Gram-negative bacteria isolated from diabetic foot ulcer infection.

Pathogen	Antibiotic (sensitive: resistant (%))								
	Ofloxacin	Ampisulbactam	Cefepime	Ceftriaxone	Ampicillin	Penicillin	Vancomycin	Gentamycin	Tetracycline
*E. coli*	0 : 1 (0 : 100)	3 : 2 (60 : 40)	5 : 3 (62.5 : 37.5)	1 : 2 (33.3 : 66.7)	1 : 0 (100 : 0)			5 : 2 (71.4 : 28.6)	
*Klebsiella*		1 : 0 (100 : 0)	4 : 3 (57.1 : 42.9)					3 : 0 (100 : 0)	
*Enterobacter*	1 : 0 (100 : 0)	1 : 0 (100 : 0)	1:1 (50 : 50)	1 : 1 (50 : 50)	1 : 0 (100 : 0)	1:1 (50 : 50)	2 : 0 (100 : 0)	2 : 0 (100 : 0)	2 : 0 (100 : 0)
*Acinetobacter*		1 : 0 (100 : 0)	4 : 0 (100 : 0)	2 : 0 (100 : 0)				1 : 0 (100 : 0)	
*P. vulgaris*		0 : 2 (0 : 100)	2 : 0 (100 : 0)		0 : 1 (0 : 100)			0 : 1 (0 : 100)	
*P. mirabilis*			3 : 0 (100 : 0)	1 : 0 (100 : 0)					
*Pseudomonas*			2 : 0 (100 : 0)					1 : 0 (100 : 0)	
*Citrobacter*			1 : 0 (100 : 0)					1 : 0 (100 : 0)	
*Fluxobacteria*		1 : 0 (100 : 0)							

S: sensitive; R: resistant.

**Table 3:** Sensitivity pattern of Gram-negative bacteria isolated from diabetic foot ulcer infection.

Pathogen	Antibiotic (sensitive: resistant (%))								
	Cephalexin	Ciprofloxacin	Cotrimoxazole	Erythromycin	Amikacin	Meropenem	Imipenem	Ceftazidime	Piperacillin/tazobactam
*E. coli*		1 : 9 (10 : 90)	3 : 8 (27.3 : 72.7)		10 : 5 (66.7 : 33.3)	12 : 0 (100 : 0)	7 : 2 (77.8 : 22.2)	7 : 4 (63.6 : 36.4)	7 : 3 (70 : 30)
*Klebsiella*		4 : 2 (66.7 : 33.3)	2 : 4 (33.3 : 66.7)		9 (100)	6 : 1 (87.7 : 14.3)	6 : 0 (100 : 0)	4 : 3 (57.1 : 42.9)	7 : 0 (100 : 0)
*Enterobacter*	1 : 0 (100 : 0)	1 : 2 (33.3 : 66.7)	3 : 0 (100 : 0)	1 : 0 (100 : 0)	1 : 2 (33.3 : 66.7)	2 : 1 (66.7 : 33.3)	2 : 0 (100 : 0)	1 : 1 (50 : 50)	1 : 1 (50 : 50)
*Acinetobacter*		4 : 0 (100 : 0)	2 : 0 (100 : 0)		1 : 3 (25 : 75)	4 : 0 (100 : 0)	1 : 0 (100 : 0)	2 : 0 (100 : 0)	3 : 0 (100 : 0)
*P. vulgaris*		0 : 2 (0 : 100)	0 : 1 (100)		3 (100)		2 : 0 (100 : 0)	1 : 0 (100 : 0)	0 : 3 (0 : 100)
*P. mirabilis*		0 : 2 (0 : 100)	0 : 1 (100)		2 : 1 (66.7 : 33.3)		2 : 0 (100 : 0)	1 : 0 (100 : 0)	1 : 2 (66.7)
*Pseudomonas*			0 : 1 (100)		2 (100)			1 : 0 (100 : 0)	0 : 2 (0 : 100)
*Citrobacter*									
*Fluxobacteria*		0 : 1 (0 : 100)					1 : 0 (100 : 0)		

S: sensitive; R: resistant.

**Table 4:** Sensitivity pattern of Gram-positive bacteria isolated from diabetic foot ulcer infection.

Pathogen	Antibiotic (sensitive: resistant (%))									
	Linezolid	Doxycycline	Amoxiclav	Ampicillin	Penicillin	Vancomycin	Gentamycin	Rifampin	Tetracycline	Cephalexin
*Enterococci*	6 : 2 (75 : 25)			8 : 3 (72.727.3)	4 : 1 (80 : 20)	11 : 2 (84.6 : 15.4)	11 : 0 (100 : 0)	2 : 0 (100 : 0)	2 : 11 (15.4 : 84.6)	1 : 0 (100 : 0)
*Staphylococcus aureus*	1 : 0 (100 : 0)	1 : 2 (33.3 : 66.7)				2 : 0 (100 : 0)	3 : 1 (75 : 25) 1 (25)			1 : 0 (100 : 0)
*Staphylococcus epidermidis*		2 : 0 (100 : 0)		0 : 1 (0 : 100)		2 : 0 (100 : 0)	2 : 1 (66.7 : 33.3)			
*Streptococcus viridans*	1 : 0 (100 : 0)			0 : 1 (0 : 100)	0 : 1 (100)	2 : 0 (100 : 0)		1 : 1 (50 : 50)	0 : 2 (0 : 100)	
*Coagulase-negative Staphylococcus*	1 : 0 (100 : 0)	0 : 1 (0 : 100)	1 : 0 (100 : 0)			1 : 0 (100 : 0)	1 : 0 (100 : 0)			
*Streptococcus beta-hemolytic*	1 : 0 (100 : 0)			0 : 1 (0 : 100)		2 : 0 (100 : 0)	1 : 0 (100 : 0)		1 : 0 (100)	
*Staphylococcus beta-hemolytic*				1 : 0 (100 : 0)	1 : 0 (100)			1 : 0 (100 : 0)	1 : 0 (100)	

S: sensitive; R: resistant.

**Table 5:** Sensitivity pattern of Gram-positive bacteria isolated from diabetic foot ulcer infection.

Pathogen	Antibiotic (sensitive: resistant (%))								
	Cefixime	Ciprofloxacin	Clindamycin	Cotrimoxazole	Erythromycin	Oxacillin	Imipenem	Piperacillin/tazobactam	Cefoxitin
*Enterococci*	0 : 1 (0 : 100)	1 : 9 (10 : 90) 9 (90)		1 : 1 (50 : 50)	2 : 0 (100 : 0)			1 : 0 (100 : 0)	
*Staphylococcus aureus*		2 : 3 (40 : 60)	3 : 4 (42.9 : 57.1)	2 : 1 (66.7 : 33.3)	3 : 3 (50 : 50)	5 : 2 (71.4 : 28.6)			1 : 0 (100 : 0)
*Staphylococcus epidermidis*			0 : 4 (0 : 100)	2 : 0 (100 : 0)	0 : 4 (0 : 100)	1:2 (33.3 : 66.7)			1 : 1 (50 : 50)
*Streptococcus viridans*		0 : 1 (0 : 100)							
*Coagulase-negative Staphylococcus*			0 : 2 (0 : 100)		0 : 2 (0 : 100)	0 : 1 (0 : 100)	1 : 0 (100 : 0)		1 : 0 (100 : 0)
*Streptococcus beta-hemolytic*		0 : 1 (0 : 100)							
*Staphylococcus beta-hemolytic*				0 : 1 (0 : 100)					

S: sensitive; R: resistant.

## Data Availability

SPSS data of the participant can be requested from the authors. The data used to support the findings of the study are available from the corresponding author upon request.
